# Using the Objective Structured Teaching Examination to Improve the Clinical Teaching Efficacy of Clinical Preceptors: A Quasi-Experimental Study

**DOI:** 10.1097/jnr.0000000000000726

**Published:** 2026-02-04

**Authors:** Ya-Wen LEE, Wan-Ru HUANG, Tsen-En CHAO, Yung-Sung WEN, Yu-Jun CHANG, Chih-Hao LIN

**Affiliations:** 1 Department of Nursing, Changhua Christian Hospital, Taiwan; 2 Graduate Institute of Clinical Nursing, College of Medicine, National Chung Hsing University, Taiwan; 3 Center of Faculty Development, Changhua Christian Hospital, Taiwan; 4 Big Data Center, Changhua Christian Hospital, Taiwan

**Keywords:** Objective Structured Teaching Examination (OSTE), clinical preceptors, nursing education, teaching competencies, quasi-experimental study

## Abstract

**Background:**

Although clinical preceptors (CPs) are vital to the retention of new nurses, evaluations of post-training changes in their teaching behavior are limited. The Objective Structured Teaching Examination (OSTE), designed to assess and enhance the instructional skills of CPs, is underutilized in nursing education.

**Purpose:**

This study was developed to evaluate the effectiveness of the OSTE in improving teaching competencies in CPs, with effectiveness tracked longitudinally at 3 and 6 months postintervention.

**Methods:**

A nonrandomized allocation, longitudinal, parallel design was used in this quasi-experimental study. One hundred twenty-two CPs working at one Taiwanese medical center were enrolled as participants and assigned to experimental (*n*=63) and control (*n*=59) groups. The experimental group completed seven OSTE sessions based on core competencies. Teaching competencies were measured using the Clinical Teaching Behavior Inventory (CTBI) at four time points: pre-OSTE, post-OSTE, and 3 and 6 months post-OSTE. Data were analyzed using generalized estimation equations.

**Results:**

The experimental group showed significant improvements in all CTBI domains immediately after the intervention (all *p* ≤ .003), with the CTBI total score remaining significantly elevated at 3 months (*p*=.026) and 6 months (*p*=.004) postintervention. The improvements in the control group were shown to be uneven and not significant until 6 months post-test. In all participants, clinical ladder level was found to relate positively to CTBI total score.

**Conclusions:**

OSTE effectively enhances teaching competencies in CPs in the short term, with these effects declining but remaining significant through at least 6 months postintervention. Ongoing training and integrated assessment tools are necessary to realize and sustain improvements in CP clinical teaching efficacy over the long term.

## Introduction

The global nursing shortage, a critical challenge to public health care exacerbated by the COVID-19 pandemic, is currently estimated at 5.9 million nurses (World Health Organization, 2020). In Taiwan, nursing turnover and vacancy rates rose, respectively, from 10.13% to 12.61% and from 4.7% to 9.05% between 2021 and 2023 (Department of Nursing and Health Care, Ministry of Health and Welfare, Taiwan, ROC, 2024). The Taiwan government has been implementing specific policies to address nursing workforce supply and demand issues since 2023 (Ministry of Health and Welfare, Taiwan, ROC, 2023). Promoting the position of clinical preceptors (CPs) is pivotal to retaining new nurses through fostering the clinical competence of these nurses and facilitating their successful integration into the nursing profession (Elbilgahy et al., 2020). Effective preceptors require robust teaching skills to support newcomers and reduce turnover (Lee et al., 2020). Although Taiwan’s Ministry of Health and Welfare has promoted teacher training certification programs since 2012 (Joint Commission of Taiwan, 2024), evaluations of these programs have generally focused on cognitive gains and satisfaction, with limited attention given to changes in actual teaching behaviors (Garavan et al., 2020). This gap underscores a need for innovative training methods to enhance these preceptors’ instructional skills and long-term impact on teaching performance.

The Objective Structured Teaching Examination (OSTE) is a structured, simulation-based method designed to assess and improve clinical teaching skills using standardized scenarios (Boillat et al., 2012; Kay et al., 2023). In the OSTE, preceptors perform teaching tasks in controlled settings, interacting with standardized students and faculty, while observers provide immediate feedback using tailored scoring sheets (Arrogante et al., 2021). This approach allows preceptors to refine their teaching and feedback techniques in a safe environment, minimizing risks to students’ learning (Abbasi et al., 2023; Fakhouri & Nunes, 2019). Rooted in experiential learning theory, the design of the OSTE facilitates skill development through practice, reflection, and feedback (Kolb, 1984). Scenarios, typically based on real-world teaching challenges such as providing feedback and modeling professional behavior, are derived from student input or focus groups (Boillat et al., 2012). Despite its current application in medical, dental, and pharmacy education, the OSTE remains underutilized in nursing, particularly as a tool for evaluating long-term changes in behavior (Kay et al., 2023).

Evidence suggests the OSTE enhances teaching competencies. In Matthews et al. (2020), medical students participating in a 1-hr OSTE workshop reported improved feedback-receiving skills (mean score increase from 28.8 to 34.5 out of 40, *p*=.013). In Shaw et al. (2016), OSTE-based programs such as the One-Minute Preceptor were shown to improve the teaching abilities of nursing instructors significantly. In Lüchinger et al. (2023), OSTE was shown to be effective in enhancing preceptor feedback skills and to positively impact clinical teaching performance. However, the focus of most studies has been on immediate outcomes rather than sustained effects (Fakhouri & Nunes, 2019). In Taiwan, Lee et al. (2020) found preceptor teaching behaviors to be influenced more significantly by hospital policies than OSTE training scores, highlighting the need to assess the OSTE’s direct impact on behavior. This study was designed to address this gap by evaluating the effectiveness of the OSTE in improving teaching competencies in CPs and tracking the sustainability of these improvements at 3- and 6-month postintervention. The findings are expected to offer novel insights into the efficacy of using the OSTE in nursing education in terms of achieving long-term improvements in CP teaching competency.

## Methods

### Design and Participation

A quasi-experimental design with nonrandomized allocation was employed to evaluate the effectiveness of using the OSTE to improve teaching competencies in CPs. One hundred twenty-two CPs were recruited from a medical center in central Taiwan between August 2019 and June 2021. The participants self-selected their group based on availability and willingness to participate in OSTE training, resulting in an experimental group (E, *n*=63), which received the OSTE training intervention, and a control group (C, *n*=59), which received standard in-service training. To ensure balanced sample sizes, the recruitment process was monitored, and adjustments were made during recruitment to achieve approximately 60 participants in each group. The self-selection approach was used to accommodate clinical schedules and enhance participation in spite of the attendant risk of selection bias, which was addressed using statistical adjustments (see section Statistical Analysis). This study was conducted in 3 phases: setup (August–October 2019, designing OSTE scenarios), implementation (June–November 2020, conducting OSTE sessions), and follow-up (September 2020–June 2021, tracking long-term outcomes).

### Intervention

The OSTE intervention was developed in line with the 12 guidelines for OSTE design proposed in Boillat et al. (2012), which emphasize using structured, simulation-based training to enhance teaching skills (Kay et al., 2023). An initial list of core teaching competencies was compiled from focus group interviews with 12 experienced preceptors, with four key areas identified: teaching attitude, communication, feedback, and evidence use. Four 20-minute scenarios were respectively designed for four stations, including: (a) Teaching Attitude and Modeling (e.g., demonstrating professionalism in a ward setting), (b) Teaching Communication (e.g., explaining procedures to a novice nurse), (c) Assessment and Feedback (e.g., providing constructive feedback on clinical skills), and (d) Evidence and Information Use (e.g., integrating research into teaching). Each station included 2 minutes for reading instructions, 8 minutes for interactive performance with a standardized student, 5 minutes for interaction with a standardized faculty member, and 5 minutes for feedback from a trained observer using a scoring sheet tailored to the scenario objectives (Boillat et al., 2012). Scenario reliability ranged from .86 to .99 (expert content validity scale), with the Kendall’s coefficient of concordance ranging from .54 to .66 and 2-week retest reliability ranging from .90 to .97. The experimental group completed seven OSTE sessions (June–November 2020), with 9–10 participants per session. The control group received standard in-service training, consisting of two 3-hr lectures on teaching strategies (e.g., lesson planning, basic feedback techniques) delivered by nursing educators without simulated scenarios or standardized feedback.

### Instruments

Demographic attributes of the participants, including age, seniority, gender, educational level, marital status, parental status, nursing clinical ladder level, work unit, and religion, were collected to characterize the sample and control for confounders.

The Clinical Teaching Behavior Inventory (CTBI), a validated tool developed by Lee-Hsieh et al. (2016), was applied in this study to assess preceptor teaching behaviors. This scale was selected based on its comprehensive coverage of preceptor behaviors and established psychometric properties in nursing contexts. The CTBI comprises 23 items across the six domains of (a) Commitment to Teaching (CT, 4 items, e.g., “I am enthusiastic about teaching”), (b) Building a Learning Atmosphere (LA, 5 items, e.g., “I create a supportive learning environment”), (c) Using Appropriate Teaching Strategies (TS, 5 items, e.g., “I adapt teaching to learners’ needs”), (d) Guiding Interprofessional Communication (IC, 3 items, e.g., “I facilitate collaboration with other professionals”), (e) Providing Feedback and Evaluation (FE, 3 items, e.g., “I provide constructive feedback”), and (f) Showing Concern and Support (CS, 3 items, e.g., “I show empathy to learners”). Items are scored on a 5-point Likert scale (1=*strongly disagree*, 5=*strongly agree*), with higher scores indicating stronger teaching competencies (range: 23–115). In the original study, the CTBI demonstrated high internal consistency (Cronbach’s alpha=.96) and construct validity via confirmatory factor analysis (χ²/*df*=2.17, CFI=0.95). In this study, the Cronbach’s alpha was .95. Authorization for use of the CTBI was obtained from the original study’s corresponding author.

### Data Collection

Data were collected at four time points to assess immediate and sustained OSTE effects: pre-OSTE (O1, May 2020), immediately post-OSTE (O2, November 2020), 3 months post-OSTE (O3, February 2021), and 6 months post-OSTE (O4, May 2021). Both groups completed the CTBI questionnaire in the same time periods to ensure consistency and control for potential temporal effects. The questionnaire was administered in paper format during scheduled staff meetings, with trained research assistants providing instructions and ensuring confidentiality. To maximize response rates, the participants were sent reminders via email and given a small incentive (NT$100 gift card) upon completion of the data collection process. The design of the CTBI and its repeated administration facilitated the detection of changes in teaching behaviors, reflecting the impact of the OSTE over time. Ethical approval was obtained from the Human Subjects Committee Research Ethics Board at the study site (IRB No. 181241). The participants provided informed consent, and data were anonymized to protect privacy.

### Statistical Analysis

Data were analyzed using IBM SPSS Statistics for Windows, Version 22.0 (IBM Corp., Armonk, NY), with *p*<.05 indicating statistical significance. Descriptive statistics were used to summarize continuous data (means, standard deviations) and categorical data (frequencies, percentages); group differences were compared using independent samples’ *t* tests, one-way analysis of variance, χ^2^ tests, or Fisher’s exact tests; and Generalized Estimating Equations (GEE) were used to evaluate the effect of the intervention on scale scores over time. An autoregressive working correlation structure was selected based on its suitability for handling longitudinal data, assuming repeated measures are more strongly correlated when closer in time.

The model included main effects for group (experimental vs. control) and time (O2, O3, and O4), along with a group-by-time interaction term to test intergroup differences in the trajectory of score changes. Furthermore, to control for potential confounding factors, the model was adjusted for several covariates, including baseline CTBI scores, work unit, nursing career ladder level, and marital status, known to potentially influence teaching behaviors (e.g., experience, workload, personal responsibilities; Liu et al., 2020). Work unit was also included due to significant baseline differences (Table 1), and nursing career ladder level and marital status were also included based on previous research showing associations with teaching competence (Lin et al., 2019). Other potential confounders (e.g., prior training, workload) were not included due to data limitations, but are noted in the study’s limitations.

**Table 1:** Participant Demographic Characteristics

Variable	Experimental Group (*n*=63)	Control Group (*n*=59)	*p*
	*n* (%)	*n* (%)	
Service length (years)			.378
<10	24 (38.1)	18 (30.5)	
≥10	39 (61.9)	41 (69.5)	
Educational level			.787
Associate degree	5 (7.9)	3 (5.1)	
Bachelor	56 (88.9)	55 (93.2)	
Graduate	2 (3.2)	1 (1.7)	
Marital status			.373
Single	31 (49.2)	22 (37.3)	
Married	30 (47.6)	35 (59.3)	
Divorced	2 (3.2)	2 (3.4)	
Children			.082
No	35 (55.6)	24 (40.7)	
Yes	28 (44.4)	35 (59.3)	
Nursing career ladder level			1.000
N1	1 (1.6)	0 (0.0)	
N2	24 (38.1)	24 (40.7)	
N3	34 (54.0)	32 (54.2)	
N4	4 (6.3)	3 (5.1)	
Work unit			.001***
Internal medicine ward	12 (19.0)	25 (42.4)	
Surgical ward/ER	14 (22.2)	20 (33.9)	
ICU/RCC	29 (46.0)	8 (13.6)	
Pediatric/obstetrics ward	5 (7.9)	3 (5.1)	
Other wards	3 (4.8)	3 (5.1)	
Religious beliefs			.334
General folk beliefs	47 (74.6)	34 (57.6)	
Taoism	9 (14.3)	11 (18.6)	
Buddhism	2 (3.2)	4 (6.8)	
Christian	2 (3.2)	5 (8.5)	
Others	3 (4.8)	5 (8.5)	

*Note. p* values were calculated using the χ^2^ test or Fisher’s exact test where appropriate. The *p* value of 1.000 for Nursing Career Ladder reflects a nearly identical distribution for this variable in both groups, confirmed by Fisher’s exact test.

ER=emergency room; ICU=intensive care unit; RCC=respiratory care center.

Minimum sample size of 45 per group was determined using G*Power 3.1 in order to detect a medium effect size (Cohen’s d) of 0.6 with 80% statistical power and a 5% type I error rate. This sample size estimate was based on findings from relevant literature on similar educational interventions used in the health care profession. For example, a medium effect size of 0.63 for perception changes was used in a study on a patient safety incident disclosure education program for nursing students (Kim & Kim, 2024). In addition, a large effect size of 1.14 was found in a study that utilized the OSTE to assess feedback-receiving skills among medical students (Matthews et al., 2020). Thus, an effect size of 0.6 was deemed in this study as reasonable and conservative. To account for potential attrition, 122 participants were recruited.

## Results

### Comparison of Demographic Characteristics and Preintervention CTBI Scores

Of the 122 participants, 63 were assigned to the experimental (OSTE) group and 59 to the control group. All of the participants were female. In the experimental group, the mean age was 34.7 years (*SD*=5.15), the mean service length was 12.6 years (*SD*=5.38), most had a bachelor’s-level education (88.9%), 31 were single (49.2%) and 30 were married (47.6%), and over half (*n*=34, 54.0%) were at the N3 nursing clinical ladder level. Also, most (*n*=29, 46.0%) worked in the intensive care unit/respiratory care center (ICU/RCC), with others working in the surgical ward/emergency room (ER, 22.2%) and internal medicine ward (19.0%). Three-quarters (74.6%) reported “general folk beliefs” as their religious affiliation. Notably, aside from a significantly higher proportion working in internal medicine wards (42.4%) in the control group leading to a significant between-group baseline difference in work unit distribution (*p*=.001; Table 1), all demographic attributes were statistically similar between the two groups (*p*>.05). Preintervention CTBI scores were significantly higher in the control group in the three domains of Guiding Interprofessional Communication (IC, 12.4 ± 1.7 vs. 11.7 ± 1.5, *p*=.017), Providing Feedback and Evaluation (FE, 12.7 ± 1.6 vs. 11.8 ± 1.6, *p*=.002), and Showing Concern and Support (CS, 12.6 ± 1.6 vs. 11.9 ± 1.9, *p*=.018), while no significant between-group differences were found in the domains of Commitment to Teaching (CT), Building a Learning Atmosphere (LA), or Using Appropriate Teaching Strategies (TS; Table 2). The higher baseline IC, FE, and CS scores in the control group suggest initial differences in these competencies, which were accounted for in the statistical analyses.

**Table 2:** Between-Group Comparison of Scale Scores and Change Over Time

Dimension/Time	Experimental Group (*n*=63)		Control Group (*n*=59)		*p* ^b^
	Mean (*SD*)	*p* ^a^	Mean (*SD*)	*p* ^a^	
Committing to teaching					
1	16.6 (1.9)		17.1 (1.7)		.136
2	17.3 (2.0)	.003**	17.0 (1.9)	.503	.421
3	16.9 (2.1)	.140	17.2 (1.8)	.752	.514
4	16.9 (2.1)	.304	17.3 (1.9)	.244	.196
Building a learning atmosphere					
1	20.1 (2.8)		20.5 (2.3)		.353
2	21.1 (2.7)	<.001***	20.7 (2.2)	.348	.426
3	20.2 (2.6)	.673	20.7 (2.5)	.579	.311
4	20.5 (3.0)	.149	21.1 (2.3)	.019*	.214
Using appropriate teaching strategies					
1	19.9 (2.6)		20.5 (2.3)		.156
2	21.4 (2.7)	<.001***	20.6 (2.3)	.608	.082
3	20.5 (2.5)	.036*	21.1 (2.6)	.058	.200
4	20.7 (2.6)	.006**	21.5 (2.6)	.001**	.129
Guiding interprofessional communication					
1	11.7 (1.5)		12.4 (1.7)		.017*
2	12.6 (1.9)	<.001***	12.3 (1.5)	.595	.331
3	12.1 (1.6)	.082	12.7 (1.6)	.076	.029
4	12.3 (1.6)	.003**	12.7 (1.8)	.225	.211
Providing feedback and evaluation					
1	11.8 (1.6)		12.7 (1.6)		.002**
2	12.8 (1.5)	<.001***	12.7 (1.5)	.844	.773
3	12.3 (1.8)	.010*	12.9 (1.6)	.438	.053
4	12.5 (1.7)	<.001***	13.1 (1.6)	.034*	.061
Showing concern and support					
1	11.9 (1.9)		12.6 (1.6)		.018*
2	12.8 (1.8)	<.001***	12.5 (1.6)	.478	.359
3	12.4 (1.9)	.031*	12.8 (1.8)	.505	.320
4	12.4 (1.9)	.025*	13.1 (1.7)	.003**	.056
Total scores					
1	91.9 (10.6)		95.9 (9.6)		.034*
2	98.0 (11.2)	<.001***	95.9 (9.6)	.966	.270
3	94.4 (11.0)	.026*	97.3 (10.4)	.189	.141
4	95.3 (11.7)	.004**	98.7 (10.7)	.005**	.098

*Note.* Time points: 1=baseline (O1); 2=immediately postintervention (O2); 3=3-month follow-up (O3); 4=6-month follow-up (O4).

^a^ *p*-value from GEE for within-group change compared with baseline (Time 1). ^b^ *p*-value from independent *t*-test for comparison between groups at each time point. Please refer to Table 4 for the multivariable GEE analysis incorporating group × time interaction.

*p<.05. **p<.01. ***p<.001.

### CTBI Domain Rankings and Overall Changes Over Time

To provide context for competency strengths and weaknesses, preintervention CTBI domain scores were ranked based on mean values across both groups. The highest-scoring domain was Using Appropriate Teaching Strategies (TS, mean=20.2 ± 2.5), followed by Building a Learning Atmosphere (LA, 20.3 ± 2.6), Commitment to Teaching (CT, 16.8 ± 1.8), Providing Feedback and Evaluation (FE, 12.2 ± 1.6), Showing Concern and Support (CS, 12.2 ± 1.8), and Guiding Interprofessional Communication (IC, 12.0 ± 1.6). The lower scores for IC suggest it as a priority area for improvement. Immediately post-OSTE (O2), the experimental group showed significant improvements across all six CTBI domains, with the Committing to Teaching (CT) domain most significantly improved (*p*=.003), and the other five domains exhibiting highly significant gains (all *p*<.001). These improvements persisted, although at slightly reduced levels, at both 3 (O3) and 6 (O4) months postintervention (*p*<.05; Table 2). Specifically, the total CTBI score for the experimental group rose from 91.9 ± 10.6 (O1) to 98.0 ± 11.2 (O2, *p*<.001), with sustained increases at 94.4 ± 11.0 (O3, *p*=.026) and 95.3 ± 11.7 (O4, *p*=.004). In contrast, the control group showed significant improvement only at 6 months (O4), with the total CTBI score increasing to 98.7 ± 10.7 (*p*=.005). This improvement was driven by gains in several subscales, including LA (21.1 ± 2.3, *p*=.019), TS (21.5 ± 2.6, *p*=.001), FE (13.1 ± 1.6, *p*=.034), and CS (13.1 ± 1.7, *p*=.003), with no significant changes recorded at either O2 or O3 (*p*>.05). These findings highlight immediate and sustained positive impact of the OSTE on teaching competencies.

### Progress in CTBI Domains Across Time Points

The CTBI scores in the experimental group peaked immediately post-OSTE (O2), with significant improvements across all domains compared to baseline (O1): CT increased by 0.7 ± 1.7 (*p*=.003), LA by 1.0 ± 1.9 (*p*<.001), TS by 1.5 ± 2.6 (*p*<.001), IC by 0.9 ± 1.8 (*p*<.001), FE by 1.0 ± 1.6 (*p*<.001), and CS by 0.9 ± 1.5 (*p*<.001), yielding a mean total score increase of 6.1 ± 8.8 (*p*<.001). At 3 months (O3), the improvements remained significant for TS (20.5 ± 2.5, *p*=.036), FE (12.3 ± 1.8, *p*=.010), CS (12.4 ± 1.9, *p*=.031), and mean total score (94.4 ± 11.0, *p*=.026), but diminished for CT, LA, and IC (*p*>.05). By 6 months (O4), the improvements remained significant for TS (20.7 ± 2.6, *p*=.006), IC (12.3 ± 1.6, *p*=.003), FE (12.5 ± 1.7, *p*<.001), CS (12.4 ± 1.9, *p*=.025), and mean total score (95.3 ± 11.7, *p*=.004). The highest mean scores were achieved in the control group at O4, with the improvements all relatively modest. Compared with baseline, mean scores at O4 had increased as follows: LA by 0.6 ± 2.0 (*p*=.019), TS by 1.0 ± 2.3 (*p*=.001), FE by 0.5 ± 1.4 (*p*=.034), CS by 0.4 ± 1.1 (*p*=.003), and mean total score by 2.8 ± 7.9 (*p*=.005), with no significant changes in CT (0.2 ± 1.6, *p*=.244) or IC (0.3 ± 1.7, *p*=.225). The larger and more immediate improvements in the experimental group underscore the effectiveness of the OSTE, particularly in the IC, FE, and CS domains, which showed sustained gains despite initial control group advantages.

### Subgroup Differences in CTBI Score Improvements

Subgroup analyses revealed that the effectiveness of the OSTE intervention varied across different preceptor characteristics (Table 3). Two main patterns emerged. First, professional experience appeared to enhance intervention efficacy, as participants at higher nursing clinical ladder levels (N3/N4) showed significantly greater improvements in guiding interprofessional communication (*p*=.045).

**Table 3:** Comparison of Mean Improvements in Clinical Teaching Behavior Inventory Scores Across Subgroups

Variable	△Committing to Teaching (CT)		△Building a Learning Atmosphere (LA)		△Using Appropriate Teaching Strategies (TS)		△Guiding Interprofessional Communication (IC)		△Providing Feedback and Evaluation (FE)		△Showing Concern and Support (CS)	
	Mean (*SD*)	*p*	Mean (*SD*)	*p*	Mean (*SD*)	*p*	Mean (*SD*)	*p*	Mean (*SD*)	*p*	Mean (*SD*)	*p*
Group		.048*		.380		.065		.008**		<.001***		.001***
Experimental group	0.41 (1.84)		0.50 (2.11)		1.01 (2.53)		0.62 (1.74)		0.77 (1.57)		0.67 (1.80)	
Control group	0.06 (1.60)		0.32 (1.93)		0.55 (2.17)		0.18 (1.50)		0.19 (1.47)		0.14 (1.20)	
Service length (years)		.150		.070		.054		.688		.301		.359
<10	0.44 (2.03)		0.69 (2.21)		1.13 (2.66)		0.36 (1.80)		0.61 (1.72)		0.52 (1.64)	
≥10	0.14 (1.55)		0.27 (1.91)		0.60 (2.18)		0.43 (1.55)		0.43 (1.46)		0.36 (1.51)	
Educational level		.577		.531		.472		.441		.033		.114
Associate degree	−0.04 (1.30)		0.38 (1.64)		1.21 (2.55)		0.25 (1.15)		0.13 (1.36)		0.17 (1.27)	
Bachelor	0.27 (1.78)		0.44 (2.07)		0.77 (2.38)		0.44 (1.69)		0.55 (1.56)		0.46 (1.59)	
Graduate	−0.11 (0.33)		−0.33 (0.87)		0.11 (0.93)		−0.22 (0.44)		-0.67 (1.00)		-0.56 (0.73)	
Marital status		.171		.121		.028*		<.001**		.011*		.064
Single	0.23 (1.84)		0.50 (2.12)		0.52 (2.51)		0.32 (1.64)		0.52 (1.44)		0.20 (1.67)	
Married	0.30 (1.62)		0.42 (1.95)		1.07 (2.17)		0.59 (1.53)		0.54 (1.57)		0.59 (1.44)	
Divorced	−0.67 (1.97)		−0.75 (1.71)		−0.25 (2.99)		−1.50 (2.11)		-0.83 (2.12)		0.33 (1.67)	
Children		.784		.558		.113		.832		.777		.068
No	0.25 (1.82)		0.48 (2.01)		0.58 (2.55)		0.43 (1.84)		0.51 (1.63)		0.38 (1.68)	
Yes	0.20 (1.66)		0.35 (2.06)		0.98 (2.19)		0.39 (1.45)		0.47 (1.48)		0.45 (1.45)	
Nursing career level		.232		.934		.728		.045*		.340		.068
ladder level	0.10 (1.96)		0.40 (2.25)		0.84 (2.56)		0.20 (1.62)		0.39 (1.57)		0.23 (1.73)	
	0.33 (1.56)		0.42 (1.86)		0.75 (2.24)		0.55 (1.64)		0.55 (1.54)		0.53 (1.42)	
Work unit		.002**		.016*		.035*		.500		.305		.532
Internal medicine ward	0.11 (1.52)		0.28 (1.91)		0.60 (2.28)		0.30 (1.60)		0.32 (1.53)		0.21 (1.62)	
Surgical ward/ER	0.48 (1.51)		0.52 (2.06)		1.10 (2.32)		0.58 (1.71)		0.50 (1.50)		0.47 (1.48)	
ICU/ RCC	−0.05 (1.96)		0.23 (2.16)		0.59 (2.59)		0.41 (1.62)		0.60 (1.65)		0.57 (1.62)	
Pediatric/obstetrics ward Pediatric/OBS ward	0.17 (1.93)		0.25 (1.51)		0.21 (2.00)		0.00 (1.62)		0.25 (1.42)		0.42 (1.47)	
Other wards	1.61 (1.75)		1.94 (1.80)		2.11 (1.57)		0.61 (1.61)		1.06 (1.43)		0.39 (1.33)	
Religious beliefs		.068		.461		.027*		.089		.137		.598
Non-Christian	0.28 (1.72)		0.43 (2.01)		0.88 (2.29)		0.46 (1.59)		0.52 (1.52)		0.42 (1.56)	
Christian	-0.43 (1.83)		0.10 (2.26)		−0.76 (3.11)		−0.43 (2.25)		0.00 (1.92)		0.24 (1.58)	

*Note.* △=average change score from baseline (O1). *p* values are calculated using independent-samples *t* tests or one-way analysis of variance for preliminary descriptive comparisons only. Please refer to Table 4 for multivariable GEE results that adjust for within-subject correlation and confounders. ER = emergency room; ICU = intensive care unit; RCC = respiratory care center.

*p<.05. **p<.01. ***p<.001.

Second, work unit was found to significantly influence intervention efficacy, with those in high-intensity units such as the ICU/RCC reflecting lower gains in CT (*p*=.002) and LA (*p*=.016), which may reflect the influence of work environment pressures. Other demographic factors, including marital status and educational level, were also found to have slight associations with outcomes, while years of service and parental status were not identified as significant predictors of improvement (*p*>.05).

### Effectiveness of OSTE: Multiple Regression Analysis

While the results of univariate analyses identified greater improvements in the experimental group across all CTBI domains, variations among subgroups required further assessment using multivariate analyses. As described in the Methods section, multiple regression models with generalized estimating equations (GEE) were used to control for baseline CTBI scores, work unit, nursing career ladder level, and marital status (Liu et al., 2020; Lin et al., 2019). Work unit was also included due to significant baseline differences (*p*=.001), while nursing career ladder level and marital status were included based on their established influence on teaching competence. Other potential confounders (e.g., workload, prior training) were not assessed due to data limitations. The experimental group showed significantly greater improvements than the control group immediately post-OSTE (O2) across all domains (*p*<.01; Table 4). For example, the increase in the mean total score for the experimental group was 5.88 ± 1.46 (*p*<.001) greater than the control group at O2. Furthermore, although this improvement declined at O3 and O4, it remained significantly higher than baseline (O1, *p*<.05). Notably, although mean scores in the control group increased gradually over the time points, statistical significance was not achieved until O4 (2.80 ± 0.94, *p*=.003). Work unit showed a significant effect on CT (higher mean scores for participants in noninternal medicine wards, *p*<.05) and TS (lower mean scores for participants in pediatric wards, *p*=.012). Also, higher nursing ladder levels (N3/N4) were associated with significantly greater improvements across all domains (*p*<.001), reinforcing the relative effectiveness of the OSTE among experienced preceptors. These findings provide novel evidence of OSTE’s immediate and partially sustained impact, modulated by professional level and clinical context.

**Table 4:** Results of Multiple Linear Regression Model With Generalized Estimating Equations Method on Average Score Improvement for Each Scale

Variable	△Committing to Teaching (CT)			△Building a Learning Atmosphere (LA)			△Using Appropriate Teaching Strategies (TS)			△Guiding Interprofessional Communication (IC)			△Providing Feedback and Evaluation (FE)			△Showing Concern and Support (CS)			△Total Scores				
	*B*	*SE*	*p*	*B*	*SE*	*p*	*B*	*SE*	*p*	*B*	*SE*	*p*	*B*	*SE*	*p*	*B*	*SE*	*p*	*B*	*SE*	*p*		
Intercept	4.43	1.02	<.011***	4.05	0.97	<.001***	5.77	1.11	<.001***	3.60	0.57	<.001***	5.01	0.59	<.001***	1.80	0.53	.001**	16.85	4.67	<.001***		
Group (at O2)																							
Experimental	0.77	0.30	.010*	0.95	0.35	.007**	1.39	0.39	<.001***	0.83	0.27	.002**	0.65	0.25	.009**	0.99	0.26	<.001***	5.88	1.46	<.001***		
Control	0.00			0.00			0.00			0.00			0.00			0.00			0.00				
Time (in control group)																							
O4	0.37	0.22	.089	0.39	0.24	.100	0.83	0.27	.002**	0.36	0.19	.065	0.34	0.18	.053	0.51	0.18	.004**	2.80	0.94	.003**		
O3	0.20	0.20	.317	−0.05	0.24	.835	0.46	0.27	.088	0.42	0.18	.017*	0.14	0.19	.484	0.22	0.19	.251	1.39	0.96	.147		
O2	0.00				0.00		0.00			0.00			0.00			0.00			0.00				
Interaction of group and time (progress differences between groups)																							
O4	−0.77	0.32	.015*	−0.96	0.37	.009**	−1.51	0.43	<.001***	−0.67	0.29	.019*	−0.64	0.26	.014*	−0.92	0.26	<.001***	−5.48	1.47	<.001***		
O3	−0.52	0.31	.092	−0.82	0.34	.017*	−1.38	0.44	.002**	−0.95	0.29	.001**	−0.68	0.29	.020*	−0.62	0.28	.027*	−4.96	1.52	.001**		
O2	0.00			0.00			0.00			0.00			0.00			0.00			0.00				
Score at baseline (O1)	−0.39	0.07	<.001***	−0.35	0.07	<.001***	−0.42	0.07	<.001***	−0.52	0.06	<.001***	−0.47	0.06	<.001***	−0.40	0.05	<.001***	−0.31	0.06	<.001***		
Work unit																							
Other wards	1.63	0.67	.015*	1.19	0.86	.164	0.82	0.82	.319	0.12	0.58	.835	0.36	0.50	.479	−0.01	0.56	.993	4.47	3.32	.178		
Obstetrics ward	0.46	0.39	.244	0.18	0.61	.770	0.73	0.81	.367	0.12	0.62	.844	0.11	0.48	.824	0.02	0.34	.961	1.68	2.37	.479		
RCC	−0.44	0.47	.346	−0.60	0.52	.244	−0.08	0.53	.878	−0.40	0.35	.246	−0.20	0.34	.564	−0.59	0.35	.087	−2.01	2.13	.346		
ER	0.11	0.37	.758	0.49	0.70	.482	0.09	0.67	.895	−0.02	0.32	.951	0.11	0.40	.773	0.05	0.39	.903	0.33	2.57	.899		
Pediatric ward	−0.26	0.48	.595	−0.51	0.51	.312	−1.14	0.46	.012*	−0.57	0.37	.126	0.11	0.42	.795	−0.23	0.44	.607	−2.33	2.13	.273		
ICU	−0.46	0.32	.146	−0.40	0.39	.303	−0.78	0.46	.087	−0.03	0.27	.912	−0.04	0.28	.880	−0.01	0.31	.966	−1.98	1.81	.273		
Surgical ward	0.21	0.30	.481	0.17	0.37	.649	0.28	0.43	.519	0.22	0.27	.426	0.05	0.25	.842	−0.01	0.27	.968	1.24	1.61	.442		
Internal medicine ward	0.00			0.00			0.00			0.00			0.00			0.00			0.00				
Nursing career ladder level																							
N4	2.06	0.53	<.001***	3.05	0.72	<.001***	2.88	0.74	<.001***	2.56	0.44	<.001***	1.08	0.47	.022*	2.55	0.47	<.001***	11.89	3.30	<.001***		
N3	2.21	0.34	<.001***	3.32	0.56	<.001***	3.17	0.50	<.001***	2.75	0.32	<.001***	1.19	0.30	<.001***	3.03	0.36	<.001***	13.17	2.21	<.001***		
N2	1.80	0.35	<.001***	2.96	0.56	<.001***	2.88	0.53	<.001***	2.32	0.32	<.001***	0.75	0.31	.016*	2.71	0.40	<.001***	11.28	2.24	<.001***		
N1	0.00			0.00			0.00			0.00			0.00			0.00			0.00				
Marital status																							
Single										−1.58	0.58	.006**				−0.69	0.69	.318					
Married										0.34	0.19	.082				0.47	0.20	.019*					
Divorced										0.00						0.00							

*Note.* *B*=regression coefficient; *SE*=standard error; RCC = respiratory care center; ER = emergency room; ICU = intensive care unit. Time points: O1=baseline; O2=immediately postintervention; O3=3-month follow-up; O4=6-month follow-up. △=change score (post-test – baseline). The “Interaction of group and time” term represents the differential progress between the experimental and control groups over time (△ Experimental group − △ Control group). Reference categories for predictors were: Marital status=divorced; Nursing career ladder level=N1; Work unit=Internal medicine ward. The GEE model was specified with an autoregressive correlation structure.

*p<.05. **p<.01. ***p<.001.

## Discussion

This quasi-experimental study was developed to evaluate the effectiveness of the OSTE in enhancing teaching competencies in clinical preceptors, with a novel focus on tracking outcomes 3 and 6 months postintervention. A teaching blueprint was developed, and four 20-minute OSTE stations, including Teaching Attitude and Modeling, Teaching Communication, Assessment and Feedback, and Evidence and Information Utilization, were designed. The experimental group, which participated in seven OSTE sessions, showed significant improvements in all CTBI domains immediately postintervention (*p*<.001) and sustained albeit diminished effects at 3 and 6 months postintervention (*p*<.05). In contrast, the control group, which participated in standard in-service lectures, exhibited modest improvements only at 6 months post-test in select domains (*p*<.05). These findings, while aligning with prior research, offer new insights into the long-term impact and varying effects on subgroups of the OSTE in the context of nursing education.

The effectiveness of the OSTE may be explained within the framework of experiential learning theory (Kolb, 1984), which posits that learning occurs through a cycle of practical experience, reflective observation, abstract conceptualization, and active experimentation. The simulated scenarios used in the OSTE provide actual teaching experiences, while standardized feedback facilitates reflection and conceptualization, enabling CPs to refine their skills for future application in practice. This theoretical framework accounts for the experimental group’s immediate improvements, as the participants actively engaged in realistic teaching tasks and received tailored feedback (Boillat et al., 2012). For example, Cerrone et al. (2017) reported that a four-station OSTE improved the communication skills of general practitioners via simulated feedback practice (*p*<.05), which echoes the gains reported in this study in the Teaching Communication and Feedback domains. McCutcheon et al. (2017) reported significant improvements in the mentoring abilities of pharmacy preceptors post-OSTE (*p*=.004), particularly in the realm of interprofessional discussion facilitation, which parallels the findings in this study in the Guiding Interprofessional Communication (IC) domain. Wu et al. (2023) observed enhanced caregiver communication post-OSTE (*p*<.001), reinforcing the applicability of the OSTE across health care profession categories. The novel contribution of this study lies in its demonstration of OSTE’s sustained, albeit reduced, effects through 6 months postintervention, addressing a gap in the research, which has focused primarily on immediate outcomes only (Fakhouri & Nunes, 2019).

The decline in CTBI scores at 3 and 6 months in the experimental group may stem from several factors. High clinical workloads, common in ICU/RCC units where 46% of the experimental group worked, may limit opportunities to apply OSTE skills, leading to skill decay (Morris, 2023). Lack of postintervention reinforcement, such as refresher workshops or peer mentoring, may have further contributed to declines, as sustained skill retention requires regular practice (Garavan et al., 2020). Moreover, hospital policies prioritizing clinical duties over teaching, as noted by Lee et al. (2020), may have constrained the ability of experimental group participants to sustain new behaviors. To address this, we propose integrating the OSTE with workplace-based assessment tools such as monthly feedback sessions with standardized checklists to reinforce skills. Regular, quarterly refresher workshops may be implemented to maintain competency gains, particularly in those working in high-workload units such as the ICU/RCC. Peer mentoring programs that pair experienced CPs with novices may also be used to foster continuous learning and accountability.

Guiding Interprofessional Communication (IC) was identified in this study as the lowest-scoring CTBI domain at baseline (mean=12.0 ± 1.6), indicating it to be a critical area for improvement. Relatively low IC scores may reflect limited prior training in interprofessional collaboration, as nursing education often emphasizes clinical skills over teamwork (Elbilgahy et al., 2020). Thus, to strengthen IC, targeted workshops should be implemented that employ interprofessional communication strategies such as role-playing with multidisciplinary teams (e.g., physicians, pharmacists). Also, simulation-based training that incorporates scenarios such as coordinating care in a multidisciplinary ward round may be used to enhance the ability of CPs to model collaboration for novices. Integrating IC-focused modules into OSTE scenarios with specific feedback on teamwork skills may further address this gap. These strategies align with McCutcheon et al. (2017), who improved pharmacy preceptors’ interprofessional mentoring using targeted OSTE stations (*p*=.001).

The results of subgroup analyses indicate that those with higher nursing clinical ladder levels (N3/N4) exhibit greater CTBI improvements, which is consistent with Lin et al. (2019), who found higher teaching evaluation scores for advanced-level nurses (*F*=5.04, *p*<.002). This suggests professional experience enhances the efficacy of the OSTE, as experienced CPs may be better able to integrate feedback into practice. Liu et al. (2020) reported similar findings, with higher clinical ladder nurses scoring higher in teaching competence (*t*=13.292, *p*<.001). However, the finding in this study of significant differences among work units (e.g., lower gains in ICU/RCC, *p*=.002) contrasts with Liu et al. (2020), who found no unit-based differences (*F*=738, *p*=.530). This discrepancy may reflect the higher ICU/RCC representation in this study (46% vs. 16.3%), where workload pressures likely constrained teaching practice. These findings offer novel insights into the contextual factors that influence the efficacy of the OSTE in practice, suggesting tailored interventions for high-workload units.

**Figure 1:**
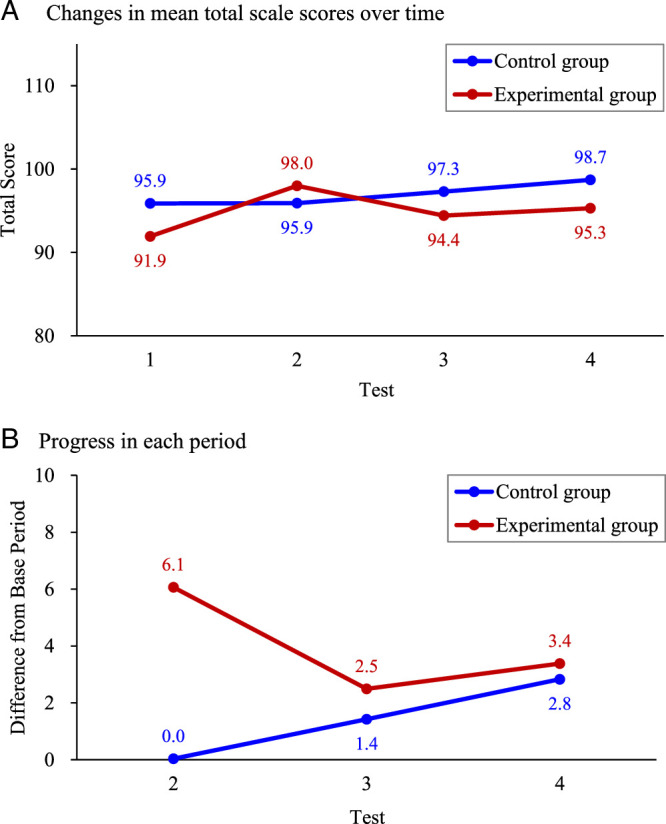
Between-Group Comparison of Clinical Teaching Behavior Inventory Scale Scores

To realize sustained OSTE benefits, practical teaching activities should be designed to reinforce competencies. For example, monthly case-based discussions in which CPs analyze real-world teaching challenges may help maintain Assessment and Feedback as well as other skills. In addition, structured peer observations, with CPs providing feedback on each other’s teaching, may enhance Teaching Attitude and Modeling. Integrating the OSTE into annual preceptor certification programs, with rotating scenarios to address IC and other domains, can help facilitate ongoing development and improvements. These activities, grounded in experiential learning, align with Wang et al. (2020), who found that preceptor teaching competence correlated positively with the core competencies of new nurses at 3 months (*r*=.22, *p*=.017). By embedding the OSTE within continuous professional development, hospitals can effectively support the long-term teaching effectiveness of clinical preceptors and ultimately improve new nurse retention rates (Figure 1).

### Limitations

This study is influenced by several limitations. The sample size (*n*=122), while sufficient for preliminary analysis, was drawn from a single medical center, which may limit generalizability to other settings. The quasi-experimental nature of this study and its use of nonrandomized allocation and a longitudinal parallel design necessitated by real-world constraints made it impossible to control fully for confounding variables, e.g., prior training and workload, which may have influenced outcomes. The follow-up periods of 3 and 6 months, while informative, may not capture effects beyond 6 months. Also, data collection was confined to a specific period (2019–2021) and location, introducing potential temporal or regional biases. Furthermore, the self-selection of groups allowed in this study may have introduced bias, although statistical adjustments were used to mitigate this. In future studies, randomized controlled designs, larger and more diverse samples, and extended follow-up periods (e.g., 12 months) should be used to validate and extend these findings. Finally, incorporating objective measures such as student evaluations of CP teaching may be employed to complement CTBI self-reports.

### Conclusions

The findings of this study provide robust evidence that the OSTE significantly enhances the teaching competencies of clinical preceptors in the short term, with partial sustainability up to at least 6 months, offering a valuable tool for nursing education. The immediate improvements realized by the experimental group across all CTBI domains, particularly in Guiding Interprofessional Communication, Feedback, and Concern and Support, demonstrate the efficacy of the OSTE in addressing critical teaching skills. However, the decline in scores found over time underscores the need for ongoing training to sustain and further increase initial gains. Higher clinical ladder CPs and those in less workload-intensive units benefited most, highlighting the influence of experience and context. To sustain improvements, integrating OSTE with continuous professional development activities such as refresher workshops and workplace-based assessments will be essential. Targeted interventions for low-scoring domains, such as interprofessional communication, may be expected to further enhance clinical preceptor effectiveness. These findings contribute novel insights into the long-term impact of the OSTE in the nursing context and support its adoption in CP training programs to improve teaching quality and, ultimately, new nurse retention rates.
